# END-TO-END VERSUS END-TO-SIDE ANASTOMOSIS IN THE TREATMENT OF ESOPHAGEAL ATRESIA OR TRACHEO-ESOPHAGEAL FISTULA

**DOI:** 10.1590/0102-6720201600010012

**Published:** 2016

**Authors:** Shahnam ASKARPOUR, Nasrollah OSTADIAN, Mehran PEYVASTEH, Mostafa ALAVI, Hazhir JAVAHERIZADEH

**Affiliations:** 1 Department of Pediatric Surgery, Imam Khomeini Hospital, Ahvaz Jundishapur University of Medical Sciences, Ahvaz, Iran; 2 Department. of Pediatric Gastroenterology, Abuzar Children's Hospital, Ahvaz Jundishapur University of Medical Sciences, Ahvaz, Iran

**Keywords:** Anastomosis, Esophageal atresia, Surgery

## Abstract

***Background*:**

Dehiscence of esophageal anastomosis is frequent and there are still controversies which type of anastomosis is preferred to diminish its incidence

***Aim*:**

To compare end-to-end anastomosis versus end-to-side anastomosis in terms of anastomotic leakage, esophageal stricture and gastroesophageal reflux symptom.

***Methods*:**

This study was carried out for two year starting from 2012. End-to-side and end-to-side anastomosis were compared in terms of anastomotic leakage, esophageal stricture, gastroesophageal reflux symptom, length of surgery and pack cell infusion.

***Results*:**

Respectively to end-to-end and end-to-side anastomosis, duration of surgery was 127.63±13.393 minutes and 130.29±10.727 minutes (p=0.353); esophageal stricture was noted in two (5.9%) and eight (21.1%) cases (p=0.09); gastroesophageal reflux disease was detected in six (15.8%) and three (8.8%) cases (p=0.485); anastomotic leakage was found in five (13.2%) and one (2.9%) cases (p=0.203); duration of neonatal intensive care unit admission was significantly shorter in end-to-end (11.05±2.438 day) compared to end-to-side anastomosis (13.88±2.306 day) (p<0.0001).

***Conclusion*:**

There were no significant differences between end-to-end and end-to-side anastomosis except for length of neonatal intensive care unit admission which was significantly shorter in end-to-end anastomosis group.

## Abstract (pt)

***Racional*:**

Deiscência de anastomose esofágica é frequente e ainda existem controvérsias qual tipo de anastomose é preferível para diminuir sua incidência.

**Objetivo:**

Comparar a anastomose terminoterminal versus a lateroterminal em termos de deiscência de anastomose, estenose de esôfago, e sintoma de refluxo gastroesofágico.

**Métodos:**

Este estudo foi realizado por dois anos a partir de 2012. Anastomoses terminoterminal e terminolateral foram comparadas em termos de deiscência de anastomose, estenose de esôfago, sintoma do refluxo gastroesofágico, duração da operação e transfusão.

**Resultados:**

Na comparação das anastomoses terminoterminal e terminolateral, respectivamente, a duração em minutos das operações foi de 127.63±13.393 e 130.29±10.727 (p=0,353); estenose esofágica foi observada em dois (5,9%) e oito (21,1%) casos (p=0,09); doença do refluxo gastroesofágico foi detectada em seis (15,8%) e três (8,8%) casos (p=0,485); deiscência de anastomose foi encontrada em cinco (13,2%) e um (2,9%) caso (p=0,203); duração do internamento na UTI neonatal foi significativamente menor na terminoterminal (11,05±2,438 dias) em comparação com terminolateral (13,88±2,306 dias, p<0,0001).

**Conclusão:**

Não houve diferença significativa entre as anastomoses terminoterminal e terminolateral, exceto para UTI neonatal que foi significativamente menor no grupo de anastomose terminoterminal.

## INTRODUCTION

Esophageal atresia has the frequency of 1 in 3500 live birth^1^^,^ 6. Survival rates of neonates who underwent end-to-side anastomosis and end-to-end anastomosis were 95% and 90% in Touloukian and Seashore^8^study. Anastomotic leakage was noted in 10% of cases whereas anastomotic stricture was seen in three cases. In 30-year follow up study by Lindahl et al, long term follow up of patients who underwent end-to-end anastomosis was similar to end-to-side anastomosis^2^. In the study by Zhang et al. end-to-end anastomosis resulted in 16% anastomotic leakage; 9% recurrent tracheo-esophageal fistula; and 10% anastomotic stricture^9^. In the study by Pietsch et al. there was no report of anastomotic leakage among 10 cases and 9% among end-to-side anastomosis^4^. In the Touloukian study, anastomotic leakage following end-to-side anastomosis (8%) were less frequent than end-to-end (13%) anastomosis^7^. Esophageal stricture was less frequent in patients who underwent end-to-side (5%) compared to patients submitted to end-to-end anastomosis (13%)^8^. 

The aim of this study was to compare end-to-end versus end-to-side anastomosis in terms of anastomotic leakage, esophageal stricture, and gastroesophageal reflux symptoms.

## METHOD

This study was approved by ethical committee of Ahvaz Jundishapur University of Medical Sciences. Informed consent was signed by parents.

It was carried out in Imam Khomeini Hospital of Ahvaz Jundishapur University of Medical Sciences, Ahvaz, Iran. In this study two groups of neonates who underwent end-to-side and end-to-end anastomosis were compared in terms of esophageal stenosis, gastroesophageal reflux presentation, length of neonatal intensive care unit admission, and mortality. Gastroesophageal reflux was evaluated clinically. Esophageal stenosis was confirmed using contrast radiography. Duration of study was two year. Seventy-two cases were enrolled and data were analyzed using SPSS version 13.0 (Chicago, IL, USA).

## RESULTS

End-to-end anastomosis was done in 38 and end-to-side in 34 cases. Recurrence rate was about zero in two groups. 

**TABLE 1:** - Comparison between two groups of patients

**p value**	**End-to-side anastomosis (n=34)**	**End-to-end anastomosis (n=38)**	
0.353	130.29±10.727	127.63±13.393	Duration of surgery(min)
0.09	8 (21.1%)	2 (5.9%)	Esophageal stricture
0.485	3 (8.8%)	6 (15.8%)	Gastroesophageal reflux
0.203	1 (2.9%)	5 (13.2%)	Anastomotic leakage
0.216	20 (41.2%)	10 (26.3%)	Tracheomalatia associated respiratory problem
<0.0001	13.88±2.306	11.05±2.438	Duration in neonatal intensive care unit
0.761	7 (20.6%)	6 (15.8%)	Mortality
0.983	12.35±3.074	12.37±3.233	Packed cell infusion (cc/kg)

Duration of hospital admission in end-to-end anastomosis group (11.05±2.438) was significantly lower than end-to-side anastomosis group (13.88±2.306, p<0.001).

## DISCUSSION

 In this study, anastomotic leakage was more frequent in end-to-end anastomosis. Brunet et al. refer anastomotic leakage significantly higher in patients who underwent end-to-side anastomosis (8/19) than the ones submitted end-to-end anastomosis (4/19). In the Touloukian and Seashore papers, anastomotic leakage was found in 5% of patients who underwent end-to-side compared to 13% submitted to end-to-end anastomosis^8^. There are differences between the results of these studies. The major difference may be related to difference in duration of follow up. 

Neonates anastomotic leakage was more frequent in patients who underwent end-to-end anastomosis compared to end-to-side. In 25-year follow up Poenaru et al enrolling 111 neonates with esophageal atresia, in 74 submitted to end-to-end anastomosis seven (9.5%) developed anastomotic leakage^5^. Of 37 neonates who underwent end-to-side anastomosis four (10.8%) had it^4^. In Pietsch et al. paper, none of 10 neonates who underwent end-to-end anastomosis developed anastomotic leakage. Of 42 neonates who underwent end-to-side anastomotic leakage was present in 9% of the cases^4^.

Gastrointestinal reflux was noted in four (10.5%) of cases in Touloukian study^7^. In this study, gastrointestinal reflux was present in 8.8% of cases which is slightly lower than Touloukian report^7^. However, duration of follow up in this study was shorter than related by these authors^7^; also, gastroesophageal reflux was more frequent in cases underwent end-to-side anastomosis compared to end-to-end. The results here observed were similar to Touloukian and Seashore study^8^.

In previous studies, type of anastomosis (end-to-side or end-to-end) had no significant difference between survivors or not after treatment of esophageal atresia^3^.

Esophageal stricture and leakage were less frequent in end-to-end anastomosis. As mentioned above, there are some differences among results of studies. They may be related to follow up duration, surgeon experience, and neonatal care after surgery. 

The limitations of this paper is that it was done in a single center and with relatively short follow up. Another multicenter study with longer follow up is recommended.

## CONCLUSION

There was no significant difference between end-to-end and end-to-side anastomosis, except for length of neonatal intensive care unit admission which was significantly shorter in end-to-end anastomosis group. 
